# Expanding the Regulatory Repertoire of GEFs

**DOI:** 10.1371/journal.pbio.1001654

**Published:** 2013-09-10

**Authors:** Richard Robinson

**Affiliations:** Freelance Science Writer, Sherborn, Massachusetts, United States of America

Small GTPases comprise a large and heterogeneous group of regulatory proteins that act throughout the cell as on/off switches, orchestrating processes as diverse as endocytosis at the plasma membrane to protein export from the nucleus. GTPases themselves are controlled by proteins called guanine nucleotide exchange factors (GEFs). Upon binding a GEF, the GTPase releases a molecule of GDP and picks up a GTP from the cytosol, inducing a conformational change in the GTPase that allows it to switch on its target protein. GTPases by themselves are relatively indiscriminate, largely relying on GEFs to determine the location and timing of their activity. Thus, understanding the factors that determine where and when a GEF binds to its GTPase is critical for understanding a wide range of transport and signaling activities in the cell.

**Figure:**
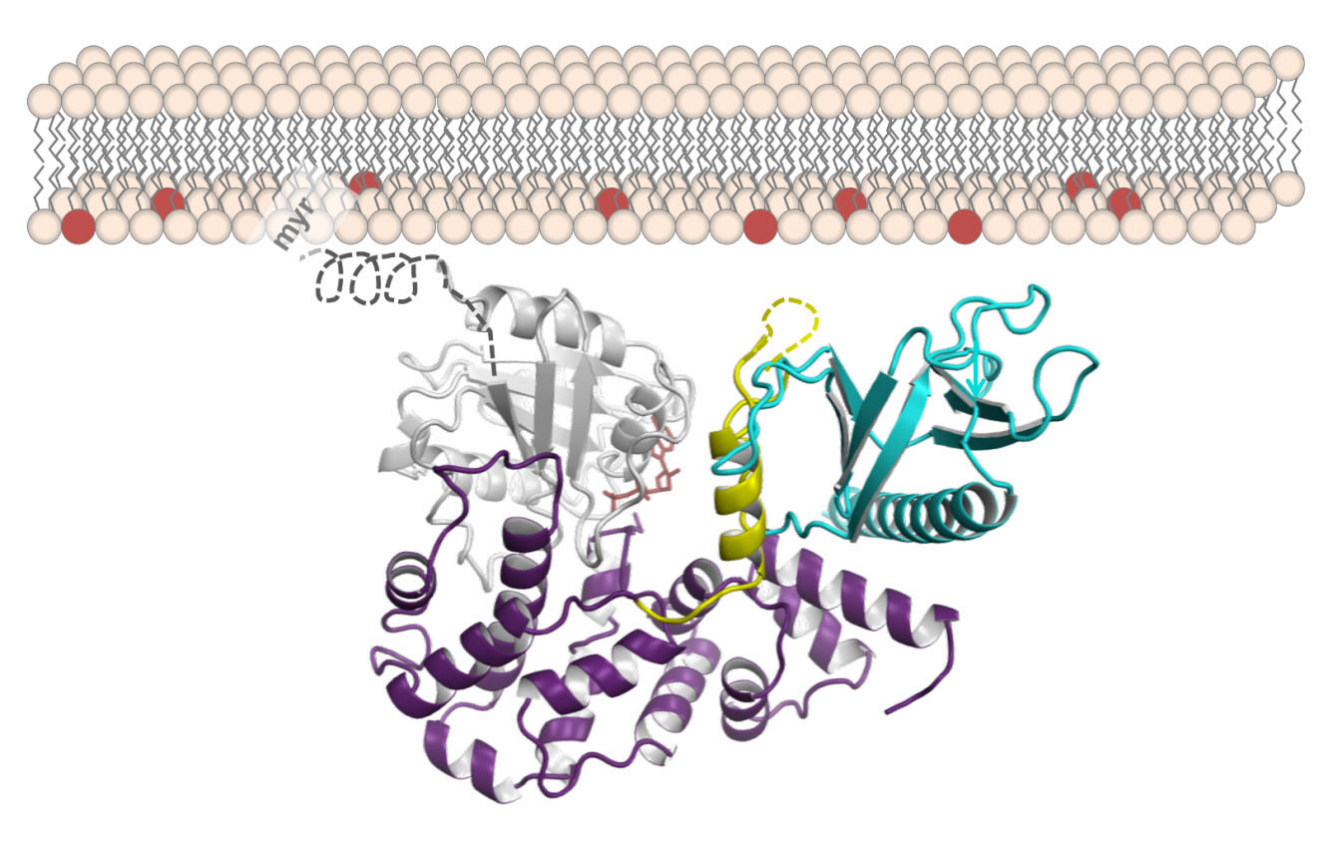
Model of the guanine nucleotide exchange factor (GEF) BRAG2 bound to membrane-anchored Arf GTPase. Cyan – BRAG2 PH domain, yellow – linker, purple – BRAG2 Sec7 domain, gray – Arf.

GTPases of the Arf class regulate most intracellular membrane trafficking, and their activity is confined to membranes by the GEFs they bind to. For one group of Arf-GEFs called cytohesins, detailed study has shown that binding specificity is controlled in part by two key factors. First, a positively charged lipid-binding pocket in the so-called PH domain attracts only a small number of specific, negatively charged phosphatidylinositides, a type of membrane lipid. Second, cytohesins in solution are auto-inhibited by an interaction between the PH domain and a catalytic domain called Sec7; membrane binding undoes that interaction, liberating the GEF to act on its target Arf. This process is further amplified by a positive feedback loop whereby freshly produced Arf-GTP binds to the PH domain.

But there are other Arf-GEFs about which little is known, including BRAG2, which has critical functions in multiple processes, such as muscle fiber development and breast tumor invasion. In a new study, Kaheina Aizel, Mahel Zeghouf, Jacqueline Cherfils, and colleagues show that the factors that control the activity of this Arf-GEF are quite different from those of the cytohesins, expanding our broader understanding of this important class of regulatory molecules.

The authors began by measuring BRAG2's activity in solution, and found that, unlike cytohesin, there was no evidence of auto-inhibition. The explanation came when they determined the crystal structure; an expansion of the PH domain in BRAG2 allowed it to establish contacts elsewhere on the protein surface, and prevented it from blocking the catalytic activity of the Sec7 domain.

This change in structure had an impact on BRAG2's exchange efficiency when paired with Arf1. The enlarged non-canonical PH domain increased the exchange activity of the Sec7 domain ten-fold compared to the Sec7 domain alone. And while BRAG2 could operate in solution, its efficiency was increased 160-fold when bound to a membrane. Interestingly, these effects were cumulative, so that the full, membrane-bound BRAG2 was more than 2000 times as efficient at promoting nucleotide exchange compared to the protein in solution without the PH domain. In further contrast, BRAG2 was not regulated by a positive feedback loop.

Next, they turned to the amino acid sequence within the catalytic pocket. They found that a positively charged lysine seen in cytohesins had been replaced by a negatively charged glutamic acid, meaning the pocket should not attract phosphatidylinositides. BRAG2 did attract negatively charged lipids, but did so through a much larger positively charged surface around the periphery of the binding pocket. As a consequence, it was much less discriminating in its choice of lipid partners, and attracted a much wider diversity of lipids compared to cytohesins, including multiple phosphatidylinositides as well as phosphatidylserine.

In sum, this study demonstrates that the regulation and lipid specificity of BRAG2 differs markedly from cytohesins, at once identifying a new regulatory scheme for GEFs and suggesting there are probably others yet to be discovered. And by shedding light on how this particular GEF is controlled, it may suggest new approaches for disrupting the activity of this protein in its role as a promoter of tumor invasion, or facilitating it in its role as a promoter of muscle repair.

**Aizel K, Biou V, Navaza J, Duarte LV, Campanacci V, et al. (2013) Integrated Conformational and Lipid-Sensing Regulation of Endosomal ArfGEF BRAG2. doi:10.1371/journal.pbio.1001654**

